# A Treatise on Hygiene and Public Health

**Published:** 1893-07

**Authors:** 


A TREATISE ON HYGIENE AND PUBLIC HEALTH. Edited by Thomas Stevenson, M. D., F. R. C. P., Lond., and Shirley F. Murphy. Vol. I. Philadelphia. P. Blakiston, Son & Co. 1892.

The editors have adopted the plan of having different authors especially qualified to write upon the various subjects coming within the scope of the work. Every subject is thus thoroughly discussed and no one part of the work has been slighted. No subject pertaining to health or hygiene has been omitted, where necessary the work is fully illustrated by cuts and drawings.

We cordially recommend the work to all health officers, sanitary inspectors, hospital and school superintendents, etc.

As a reference book it should find a place in the library of the progressive physician.

